# Step-Counting Accuracy of a Commercial Smartwatch in Mild-to-Moderate PD Patients and Effect of Spatiotemporal Gait Parameters, Laterality of Symptoms, Pharmacological State, and Clinical Variables

**DOI:** 10.3390/s23010214

**Published:** 2022-12-25

**Authors:** Edoardo Bianchini, Bianca Caliò, Marika Alborghetti, Domiziana Rinaldi, Clint Hansen, Nicolas Vuillerme, Walter Maetzler, Francesco E. Pontieri

**Affiliations:** 1 Department of Neuroscience, Mental Health and Sensory Organs (NESMOS), Sapienza University of Rome, 00189 Rome, Italy; 2 Santa Lucia Foundation, IRCCS, 00179 Rome, Italy; 3 Department of Neurology, Kiel University, 24105 Kiel, Germany; 4 AGEIS, Université Grenoble Alpes, 38000 Grenoble, France; 5 LabCom Telecom4Health, Orange Labs & Université Grenoble Alpes, CNRS, Inria, Grenoble INP-UGA, 38000 Grenoble, France; 6 Institut Universitaire de France, 75005 Paris, France

**Keywords:** gait, IMU, Parkinson’s disease, sensors, smartwatch, step detection, step count, accuracy, validation

## Abstract

Commercial smartwatches could be useful for step counting and monitoring ambulatory activity. However, in Parkinson’s disease (PD) patients, an altered gait, pharmacological condition, and symptoms lateralization may affect their accuracy and potential usefulness in research and clinical routine. Steps were counted during a 6 min walk in 47 patients with PD and 47 healthy subjects (HS) wearing a Garmin Vivosmart 4 (GV4) on each wrist. Manual step counting was used as a reference. An inertial sensor (BTS G-Walk), placed on the lower back, was used to compute spatial-temporal gait parameters. Intraclass correlation coefficient (ICC) and mean absolute percentage error (MAPE) were used for accuracy evaluation and the Spearman test was used to assess the correlations between variables. The GV4 overestimated steps in PD patients with only a poor-to-moderate agreement. The OFF pharmacological state and wearing the device on the most-affected body side led to an unacceptable accuracy. The GV4 showed an excellent agreement and MAPE in HS at a self-selected speed, but an unacceptable performance at a slow speed. In PD patients, MAPE was not associated with gait parameters and clinical variables. The accuracy of commercial smartwatches for monitoring step counting might be reduced in PD patients and further influenced by the pharmacological condition and placement of the device.

## 1. Introduction

Walking is one of the most common motor activities in daily life, is essential for the correct functioning of several physiological systems, affects quality of life, and contributes to personal and social relationships [1]. Gait parameters such as a reduced step length and walking speed are useful indexes of morbidity and mortality in older adults [2]. In particular, a daily step count is rather easy to collect, and may represent a useful surrogate measure of physical and ambulatory activity that is associated with relevant clinical outcome parameters [3]. For example, previous studies associated a reduced number of steps per day with relevant pathological conditions, including cancer and cardiovascular diseases [4], obesity, major depressive disorder, diabetes and hypertension [5], and in general mortality [6,7]. As to neurodegenerative disorders, a recent report showed the link between the daily number of steps collected through a wrist-worn accelerometer and the incidence of dementia in a large cohort of patients [8].

The diffusion of wearable devices encompassing step-counting algorithms, in particular consumer-grade wearables such as smartwatches, enabled the easy collection and monitoring of daily steps for prolonged periods. Consequently, research focused on investigating the accuracies of these devices in step counting, with positive results in general [9,10]. However, step-detection algorithms are generally trained on healthy, young subjects during continuous physiological walking; therefore, performance may be reduced when applied to patients under real-word conditions and showing altered walking parameters and/or pathological gaits.

Parkinson’s disease (PD) is a neurodegenerative disease characterized by bradykinesia, rigidity, and a resting tremor [11]. Aside from these symptoms, gait disturbances and balance impairments are frequent and significantly increase the risk of falls [12], carrying heavy impacts on functional independence and quality of life. In addition to motor symptoms, a constellation of non-motor symptoms has been reported in PD patients, including cognitive impairment [13]. This is characterized by the involvement of executive functions [14], and may further predispose to gait impairment. Indeed, previous studies have demonstrated the link between dysexecutive symptoms and reduced performances in walking tasks [15]. Gait impairments in PD patients include a shuffling gait, reduced step length and automaticity, diminished arm swing, increased double-support time and cadence, as well as episodic disturbances such as freezing of gait (FOG) [16]. The potential usefulness of gait parameters, particularly step length, to monitor disease progression has been shown recently [17,18], and interest in wearable devices for the evaluation of gait impairments in PD patients has been growing rapidly [19]. A patient’s total daily step count was associated with disease severity [20], and has been used to study ambulatory activity in PD patients [21,22] and also to guide exercise to increase physical activity [23]. However, concerns have been raised about the accuracy of consumer-grade wearables for step counting in patients with PD. In particular, two studies showed positive results [24,25], whereas a third investigation showed disappointing performances for wrist-worn devices during overground walking [26]. It was hypothesized that gait alterations and motor symptoms of PD patients could hamper the performances of step detection algorithms if not adequately validated in such cohorts showing altered mobility performances. In particular, the performances of these devices are lower with a reduced gait speed [27,28,29] and increased cadence [25], which are both features of a parkinsonian gait, and during discontinuous walking [26] that likely occurs in daily living and may be exacerbated in PD patients due to reduced automaticity and episodes of FOG. Moreover, tremors and dyskinesia may lead to the overestimation of the step count [30], particularly when using wrist-worn devices, while bradykinesia and reduced arm swing may cause underestimation [26]. The side on which the device is worn might also affect device performance, which may be an issue especially in PD, a disease where the lateralization of symptoms regularly occurs. Eventually, all of these issues may vary during daytime due to fluctuations of symptoms.

To our best knowledge, no study to date investigated the accuracy of commercial smartwatches in counting steps in PD patients taking into account the side on which the watch is worn and the pharmacological state of the patients. Similarly, no research studied the relationships between step-count error and spatiotemporal gait parameters, executive functions, and clinical scores in people with PD.

## 2. Materials and Methods

This was a cross-sectional study aimed at investigating the accuracy of a consumer-grade smartwatch in counting steps in PD patients in comparison with a reference population of healthy subjects (HS). Moreover, we explored the possible effects of side on which the watch was worn, the pharmacological state, and the association between step-count error and spatiotemporal gait parameters, executive functions, and clinical scores.

This study was performed in accordance with the ethical standards as laid down in the 1964 Declaration of Helsinki and its later amendments. Approval was granted by the local Ethical Committee. Data collection and processing followed the current European regulation for data protection. All participants provided written informed consent before the start of measurements.

Participants were longitudinally recruited in the Movement Disorder Outpatient Service of the Sant’Andrea University Hospital, Rome, Italy, in the period between June 2021 and September 2022. The inclusion criteria were: (i) age 18 years or older; (ii) ability to walk independently without walking aids; (iii) ability to perform the experimental procedure. For PD patients, the following adjunctive criteria were applied: (iv) diagnosis of idiopathic PD according to MDS criteria [31]; (v) disease stage <4 according to the modified Hoehn & Yahr (H&Y) scale [32]. The exclusion criteria were: (i) cognitive impairment as defined by a Mini Mental State Examination (MMSE) score <24; (ii) orthopedic, rheumatologic, or systemic conditions affecting mobility as judged by the assessor. For PD patients, the following adjunctive criterion was applied: (iii) presence of FOG as defined by a score >1 at item 2.13 of the Movement Disorder Society Unified Parkinson’s Disease Rating Scale part II (MDS-UPDRS).

Participants were evaluated in the morning, and they were asked to perform a 6 min walking task (6MWT) on a 25 m indoor corridor while wearing two Garmin Vivosmart 4 (GV4) smartwatches, one at each wrist. The smartwatches were configured according to the producer’s recommendations by indicating the patient’s age, height, weight, and the wrist on which the smartwatch was worn (i.e., left or right). As a reference measure, steps were counted manually by a dedicated and trained operator using a digital click counter. Participants were also asked to wear a research-grade wearable inertial-motion unit sensor (BTS G-walk, BTS Engineering Inc., Milan) at their lower back (L5) through an elastic belt to compute spatial-temporal gait parameters. HS performed the 6MWT at both a self-selected speed and at a gait speed self-perceived as “slow”. PD patients performed the test only at self-selected speed before the usual morning dose of L-DOPA (OFF) and at least 1 h after the dose administration (ON). If the patients were not in treatment with L-DOPA, they were evaluated only in the ON condition.

Demographics (age, sex) and anthropometric measures (weight, height, BMI) were collected for all participants. For PD patients, disease duration, disease stage according to the H&Y scale, and the Levodopa equivalent daily dose (LEDD) were also collected. The MDS-UPDRS [33] parts I–II were used to assess the impact of motor and non-motor symptoms on daily life. The Parkinson’s Disease Questionnaire-39 (PDQ-39) was used to evaluate the quality of life of the patients [34]. The functional independence measure (FIM) was used to assess functional independence in daily life activities [35]. The MDS-UPDRS part III and the word color Stroop test (WCST) [36] were administered both in the OFF and ON conditions to evaluate the motor symptoms’ severity and executive cognitive functions of enrolled patients, respectively.

Steps were collected from both smartwatches at the end of each walking trial. For PD patients, steps counted by the smartwatch worn on the most-affected side (MA) and on the least-affected side (LA) were recorded. To compare PD patients and HS, the average of steps counted by the smartwatches worn on right and left wrists were calculated. Spatiotemporal gait parameters from G-Walk were extracted using the software G-Studio (BTS Engineering Inc., Milan) [37]. These included the total traveled distance (m), gait speed (m/s), cadence (steps/min), and stride length (m). Gait parameters were the average across the 6 min walking trial. The first and last 25 m walking bouts were excluded from the analysis by the software as the default setting. From the number of steps counted by GV4 and manually, the mean percentage error (MPE) and the mean absolute percentage error (MAPE) were calculated as follows:

$$\mathrm{MPE}=\frac{\left(\mathrm{steps}\;\mathrm{counted}\;\mathrm{manually}\right)-\left(\mathrm{steps}\;\mathrm{counted}\;\mathrm{by}\;\mathrm{smartwatch}\right)}{\left(\mathrm{steps}\;\mathrm{counted}\;\mathrm{manually}\right)}\times 100\;\mathrm{MAPE}=\frac{\left|\left(\mathrm{steps}\;\mathrm{counted}\;\mathrm{manually}\right)-\left(\mathrm{steps}\;\mathrm{counted}\;\mathrm{by}\;\mathrm{smartwatch}\right)\right|}{\left(\mathrm{steps}\;\mathrm{counted}\;\mathrm{manually}\right)}\times 100$$

The MPE was used to evaluate the direction-of-error of the smartwatch compared with manual counting. A negative value indicated an overestimate of counting and vice-versa. The MAPE was used to assess the magnitude-of-error of GV4 in step counting compared with manual counting.

Recently, the Consumer Technology Association Health and Fitness Technology Subcommittee recommended a MAPE threshold within 10% for wearable technology in step counting [38]. On this basis, in our study, we set the MAPE cut-off for acceptability at 10% considering the upper bound of the 95% confidence interval (CI). In addition to the acceptability threshold, according to previous studies [39], we set a cut-off for optimal performance at a MAPE < 5%.

Statistical analyses were performed using JASP v0.16.3 (JASP Team, University of Amsterdam, Amsterdam, The Netherlands), R v4.0.3, and RStudio v2022.07.1 + 554 for Windows (R Foundation for Statistical Computing, Vienna, Austria). Descriptive statistics were calculated for the examined variables. To assess the agreement between the number of steps counted by GV4 and manual counting (gold standard), the intraclass correlation coefficient (ICC) [1,2] was used. The following reference cut-off values for the ICC interpretation were used [40]: Excellent: >0.90; Good: 0.75–0.90; Moderate: 0.50–0.75; Poor: <0.50. The Mann–Whitney test was used to compare the MPE and MAPE between the general PD population and HS at a self-selected speed (HS-SE). In PD patients, the average of the values collected in ON and OFF was used for the comparison. The Wilcoxon test was performed to assess the difference between the MA and LA and between ON and OFF in the MAPE and MPE of the PD patients. Spearman’s rho correlation coefficient was used to test the correlation between the MAPE and gait parameters in walking trials from the overall population, as well as from HS and PD patients. Spearman’s coefficient was also used to assess the correlation between the MAPE and clinical variables in PD walking trials. To control error from multiple tests, Bonferroni’s correction was performed. The significance threshold was set at α < 0.05. All data were reported as the mean ± SD or median (Q1–Q3) for numerical data and N (%) for categorical variables.

## 3. Results

A total of 52 PD patients and 47 healthy participants were enrolled in this study. Five PD patients were excluded from the analysis due to technical problems. Among the PD patients, 36 were evaluated in ON and 34 in OFF (with 23 patients evaluated in both conditions). Relevant demographic and clinical parameters are shown in Table 1. More details on clinical scores and gait parameters are presented in Supplementary Tables S1 and S2. 

In PD patients, GV4 demonstrated a poor-to-moderate ICC with manual step counting in the overall group and in the ON state, irrespective of the side where the watch was worn. In the OFF state, GV4 demonstrated a poor ICC, particularly when the watch was worn on the most-affected side (Table 2).

**Figure 1:**
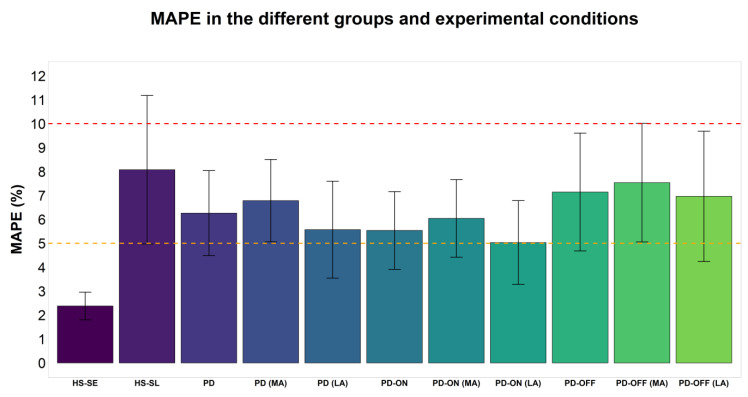
MAPE between the number of steps recorded by GV4 and manual counting in the two groups and in the different experimental conditions. In HS, data are reported for self-selected speed and slow speed. In PD patients, data are reported for the overall population, in ON, in OFF, and for the most- and least-affected body sides. Columns represent the mean of MAPE and the vertical bars represent the CI 95% upper and lower bounds. Two red and orange horizontal dashed lines were added to mark the cut-offs of 10% and 5%, respectively. HS-SE: Healthy subjects at self-selected speed; HS-SL: Healthy subjects at slow speed; LA: Least-affected side; MA: Most-affected side; MAPE: Mean absolute percentage error; PD: Parkinson’s disease.

Considering the MAPE, GV4 showed an acceptable but sub-optimal accuracy in PD patients in all conditions but wearing the device on the most-affected body side during the OFF pharmacological state. Under this latter combination of instances, the accuracy range was unacceptable (Figure 1 and Table 3). The MAPE was significantly higher when the smartwatch was worn on the most-affected compared with the least-affected body side, and in the OFF compared with the ON condition. The MPE showed negative values in all conditions, indicating an overestimation of step counting by GV4 (Table 3). In the HS population, GV4 demonstrated a good-to-excellent ICC with manual step counting while walking at a self-selected speed. On the contrary, during slow walking, GV4 demonstrated only a moderate-to-good ICC (Table 2). GV4 demonstrated optimal accuracy when walking at a self-selected speed, with a significantly lower MAPE compared with PD patients. Conversely, in slow walking, an unacceptable accuracy was achieved (Figure 1). MPE showed a positive value in this latter condition, indicating an underestimation of step counting. Details of the MAPE and MPE values are shown in Table 3. Details of the measured steps by GV4 and manual counting are shown in Supplementary Table S3.

**Figure 2:**
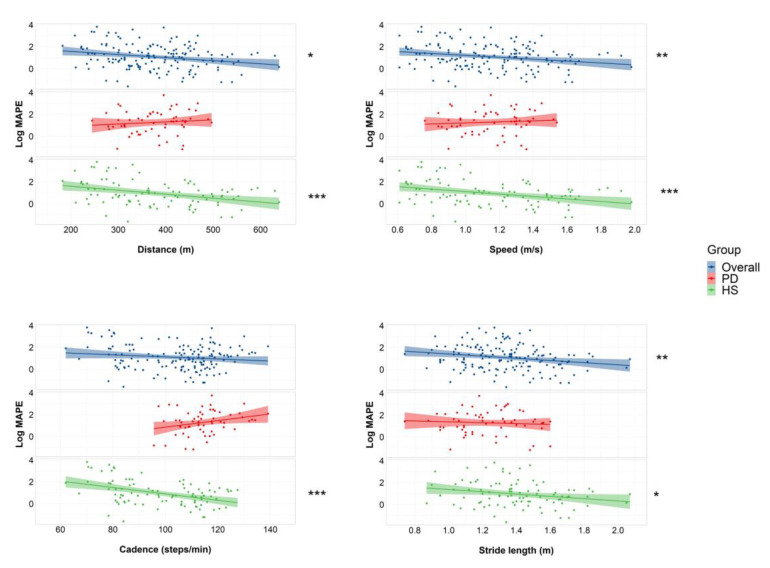
Scatterplots showing association between spatiotemporal gait parameters and MAPE. Scatterplots are presented for the overall population (all participants, PD + HS) (upper grid panels, in blue), PD patients (middle grid panels, in red), and healthy participants (bottom grid panels, in green). MAPE is reported as logarithm. A continuous line was drawn to show the linear tendency. The shaded area around the tendency line corresponds to the 95% confidence interval. Significance is reported as follows: * *p* < 0.05; ** *p* < 0.01; *** *p* < 0.001. HS: Healthy subjects; MAPE: Mean absolute percentage error; PD: Parkinson’s disease.

The Spearman test showed a weak negative correlation between the MAPE and the walked distance, gait speed, and stride length in the overall population. When considering the HS group, these findings were confirmed, and a weak negative correlation between cadence and the MAPE was also found. However, no correlation was found between the spatiotemporal gait parameters and MAPE in PD patients (Figure 2 and Table 4). Similarly, no correlations were found between other demographic or clinical variables in PD patients.

## 4. Discussion

This was a cross-sectional study investigating the accuracy of GV4 in counting steps during overground walking in PD patients in comparison with a reference population (HS) and exploring the possible effects lateralization of symptoms, pharmacological state, and the association between step-count error, spatiotemporal gait parameters, and clinical variables in PD patients.

We found that: (i) GV4 overestimated steps in PD with only a poor-to-moderate agreement; (ii) in PD patients, the OFF condition and wearing the smartwatch on the most-affected side negatively affected the step-count accuracy with an unacceptable error when combining the two factors; (iii) GV4 showed a good-to-excellent agreement and MAPE in HS at a self-selected speed, but an unacceptable performance at slow speed, and; (iv) the MAPE was associated with gait parameters in HS, but no association with gait and clinical variables was found in PD patients. These results are discussed in detail in the following.

### 4.1. Accuracy of GV4 vs. Manual Step Counting

In PD patients, GV4 showed a poor-to-moderate agreement, with an overall acceptable error range based on the a priori criterion. Conversely, in HS, GV4 showed a good-to-excellent agreement and an optimal error at a self-selected speed, but only a moderate-to-good agreement with an unacceptable error at slow speed. The sub-optimal performance of GV4 in PD patients found in our study may be due to altered gait biomechanics and the possible interference by the motor symptoms of the disease. PD gait is characterized by a reduced gait speed and step length, higher cadence, and variability [16]. Previous reports demonstrated that the accuracy of step counting by commercial smartwatches is reduced with a lower gait speed [27,28,29], higher cadence [25], and discontinuous gait [26]. The present observation of reduced accuracy during slow-speed walking (0.86 ± 0.15 m/s) as compared with self-selected-speed walking (1.44 ± 0.22 m/s) in HS is in line with this idea. Moreover, the altered gait features of a parkinsonian gait could affect the step-counting performance through a decreased signal-to-noise ratio in the accelerometer signal and a consequently reduced performance of the built-in step-detection algorithm. This is also in line with a previous study showing the low accuracy of wrist-worn smartwatches in step counting in PD patients [26]. The present results are in contrast with previous studies, suggesting the good accuracy and agreement between commercial smartwatches and reference measures in PD subjects [24,25]. In particular, Lai and collaborators [24] reported an excellent ICC (0.96–0.99) with a 0.99% error for the Garmin Vivosmart 3 smartwatch in 31 PD patients at a self-selected speed. Despite similar demographic, anthropometric, clinical, and experimental features, the authors used a different device, and no information was given on the pharmacological state or the side on which the smartphones were worn. Moreover, Lamont and colleagues [25] reported a very good ICC (0.88–0.97) and low error (<3%) for a Garmin Vivosmart HR worn on the most-affected side when compared with a research-grade device in 33 mild-to-moderate PD patients walking at a self-selected speed. However, almost half of the cases of the cohort enrolled in that study were classified as H&Y stage one, indicating an early disease stage and (mild) unilateral symptoms, which usually display less pronounced gait disturbances than our cohort, with a median disease stage of two. Moreover, a shorter and more continuous walking paradigm (2 min, 44 m walking) was applied, and a thigh-worn research-grade activity tracker (ActivPAL3) was used as a reference, without manual counting. The usefulness of the ActivePAL3 as a reference, however, might be questioned, as the underestimation of step counting has been reported in a previous study [41].

### 4.2. Effect of Side and Pharmacological State

In PD patients, step counting by GV4 was less accurate when the smartwatch was worn on the most-affected side. This finding is consistent with our hypothesis that the asymmetry of PD could affect the performance of wrist-worn smartwatches. Indeed, PD is an asymmetric disorder, usually presenting with unilateral symptoms at onset that subsequently spread to the other side, and even when the disease becomes bilateral, a degree of asymmetry is still retained [42]. Thus, bradykinesia and tremors could lead to step counting underestimation or overestimation, respectively, in a different manner according to the laterality of symptoms. With this respect, only two previous reports evaluated this aspect using two commercial smartwatches (Garmin Vivosmart HR and Fitbit Charge HR) [25] or a research-grade actigraph (ActiGraph GT3X+) [43], and the results are in contrast with ours. The study from Lamont and colleagues [25] reported no significant differences between the steps recorded on the most-affected side and the least-affected one. However, the authors only compared the absolute number of steps, and did not compare the error in step counting between the two sides. Moreover, enrolled PD patients were at an early disease stage, and were evaluated only in the ON condition. This could have masked the difference. Conversely, the study from Cederberg and collaborators [43] showed that the accuracy of the investigated device was higher when it was worn on the side most-affected by the disease. The authors suggested that the reduced movement on this side in PD patients would be advantageous for a wrist-worn detector because the most-affected side could be considered as a “non-dominant” wrist, and a previous study on healthy participants reported a better accuracy in step counting while performing daily activities when the watch was worn on the non-dominant arm [43]. However, other studies reported no effect of wrist placement on step counting in the general unimpaired population [29,44]. In addition, the explanation only considers the reduction of arm swing, and does not take into account the presence of a tremor during walking in PD patients. Indeed, approximately 70% of PD patients have a tremor, and this is usually more prominent at the upper-limb level and generally worsens during walking [45]. Moreover, in more advanced PD patients, involuntary movements (i.e., dyskinesias) are often observed [11]. Our findings support the idea that wearing a smartwatch on the most-affected side could hinder the accuracy of step counting, and the negative values of MPE indicate that the direction of error is toward an overestimation of steps, likely due to the positive motor symptoms observed in PD patients.

We also found that the pharmacological state influenced step-counting accuracy in PD patients, with a lower agreement and increased error in the OFF condition. This aspect was neglected in previous studies [24,25,26], and only one pharmacological condition was investigated [25] or it was not reported at all [24,26]. Dopaminergic treatment could influence the step-counting performance through the improvement of motor symptoms (i.e., reduced tremor and bradykinesia, increased arm swing), as well as the improvement of gait parameters. Previous research, in fact, suggested that arm swing [46], as well as gait speed and step length, may improve with L-DOPA [16], while other gait aspects, such as cadence, do not seem to respond significantly to treatment [16]. Most PD patients develop daily motor fluctuations along the disease course due to the combination of pulsating drug administration and disease progression [47]. Therefore, such different accuracies between the ON and OFF conditions may limit the reliability of commercial smartwatches concerning step counting in fluctuating PD patients, particularly when combined with the aforementioned effect of device placement. In our study, indeed, the combination of the OFF pharmacological state with the placement of the device on the most-affected side leads to an unacceptable MAPE based on the a priori criterion of 10%. To this end, it would be important for future investigations to report information about device placement and pharmacological state, and smartwatches should be worn on the side less-affected by the disease to increase the accuracy and reliability of step counting. Moreover, it would be relevant for validation studies to evaluate the device in both the ON and OFF conditions. Alternatively, a potential solution to overcome these limitations could be the development of dedicated step-detection algorithms using IMU data from smartwatches. However, this option would greatly reduce real-world applicability and hinder the benefits of using an off-the-shelf device, especially in settings where engineering and software-development expertise are not available.

### 4.3. Association of Step-Count Error, Spatiotemporal Gait Parameters, and Clinical Variables

The MAPE showed a weak correlation in the overall population and in HS with walked distance, gait speed, and stride length, whereas no correlations between the MAPE and gait variables were found in PD patients. The observation that spatial-temporal gait parameters may affect the step-counting accuracy when measured with wrist-worn sensors has already been shown in previous studies [25,27,28,29]; however, this was only assessed through discrete speed values on a treadmill [27,28,29] or discrete cadences through the use of a metronome [25], and not on a continuous range of values. The difference we found between PD patients and HS may be explained by the altered gait biomechanics in PD patients. The reduced gait speed coupled with the lower stride length may lead to a decreased signal-to-noise ratio in the accelerometer signal and a possible masking effect of the association between spatiotemporal gait parameters and the MAPE. Finally, the lack of correlation between the MAPE and clinical variables in PD patients is in contrast with our hypothesis of an error increase with a higher motor-symptoms severity and reduced executive functions. Previous studies, indeed, demonstrated that, in PD patients, 24% of variation-in-difference in daily step-counting between waist-worn and wrist-worn devices may be explained by tremor, dyskinesias, and bradykinesia, with 19% explained by tremor and dyskinesias alone [30]. An effect of executive functions on ambulation has also been reported in the general population [15] and PD patients [48], even though a recent study questioned the extent of this influence in these latter patients [49]. The results from our study suggest that PD, at least with the disease severity included in our study, is more likely to affect the step-counting performance by its presence or absence in a more dichotomous way than on a continuous range of symptoms severities.

### 4.4. Limitations

We acknowledge that this study has limitations. First, the in-lab experimental setting, consisting of a straight walking task on a 25 m walkway, is not highly representative of everyday walking behavior. Indeed, daily walking is characterized by a more discontinuous gait pattern with briefer walking bouts and more frequent turning events [50]. However, it must be noted that, under daily life conditions, a deterioration in automatic gait analysis performance likely occurs. Therefore, the results of a suboptimal accuracy in the step counting of GV4 under in-lab conditions are particularly relevant, and could point toward an even-lower performance in a real-world setting. Second, the PD patients included in our studies had, on average, relatively high levels of physical and cognitive abilities, and participants with a more severe disease stage, reporting FOG and using walking aids, were not included. This could limit the generalization of our results, and suggests that further studies enrolling participants with lower functional scores, greater disease severities, and more severe cognitive impairments are necessary. However, this might suggest a potential further deterioration in step-counting performance when applied to patients with more severe impairments. Third, the PD patients and HS were different in terms of age and anthropometric data, and this could hamper the comparison between the two populations. With this respect, however, our HS cohort has not been enrolled to perform a strict matched comparison, but rather to include a reference population more representative of the subjects on which smartwatches’ algorithms are often trained. Finally, a question might arise about the accuracy of the BTS G-Walk in measuring gait parameters in PD patients, due to the fact that a previous study [37] suggested that variables in the temporal domain (e.g., gait cycle duration, double and single support, etc.) should be used with caution due to the presence of a systematic error. However, in the present study, we did not use temporal gait parameters, and the device demonstrated a good accuracy for the included variables [37].

## 5. Conclusions

In PD patients, GV4 overestimated the number of steps with acceptable but sub-optimal accuracy. The accuracy worsened when the device was worn on the most-affected side and the patients were in the OFF state, and was unacceptable with the two element combined. These findings could be due to the altered gait biomechanics or the worsening of tremor and dyskinesias. In HS, the performance was optimal at a self-selected speed but unacceptable when walking at slow speed. The error in step counting increased with a decreasing gait speed, stride length, cadence, and walked distance in HS, while no association between gait parameters and error was found in PD patients. This could be due to the decreased signal-to-noise ratio in the accelerometer signal. Eventually, no association between the error in step counting and clinical variables was found in PD patients. Commercial smartwatches might be used to monitor steps in mild-to-moderate PD patients, but the overall reduced accuracy in PD patients must be considered together with the negative effect of the OFF pharmacological state and wearing the device on the most-affected side.

## Figures and Tables

**Table 1:** Demographic and clinical features of the enrolled population. BMI: body mass index; H&Y: Hohen and Yahr disease stage; HS: healthy subjects; LEDD: Levodopa equivalent daily dose; PD: Parkinson’s disease.

	PD (N = 47)	HS (N = 47)
**Age (years)**	66.3 ± 8.2	42.0 ± 15.3
**Females**	14 (33%)	33 (63%)
**Weight (kg)**	77.6 ± 12.2	71.0 ± 13.8
**Height (cm)**	173.9 ± 8.7	170.6 ± 7.6
**BMI**	25.6 ± 2.9	24.4 ± 4.3
**H&Y**	2 (2–2)	-
**Disease duration (years)**	6.1 ± 5.1	-
**LEDD (mg)**	576 ± 317	-

**Table 2:** Intraclass correlation coefficient between the number of steps recorded by GV4 and manual counting. ICC is reported as point estimate (CI 95%). The values are reported for at-selected speed and slow speed for healthy participants. In PD patients, values are reported in the overall population, in ON, in OFF, and for the most- and least-affected sides. HS-SE: Healthy subjects at self-selected speed; HS-SL: Healthy subjects at slow speed; ICC: Intraclass correlation coefficient. LA: Least-affected side; MA: Most-affected side; PD: Parkinson’s disease.

	HS-SE	HS-SL	PD	PD ON	PD OFF
**ICC** [1,2]	0.901 (0.856–0.933)	0.686 (0.535–0.789)	0.658 (0.306–0.825)	0.749 (0.407–0.881)	0.305 (0.039–0.535)
**ICC** [1,2] **(MA)**	-	-	0.644 (0.247–0.824)	0.719 (0.424–0.856)	0.184 (−0.085–0.434)
**ICC** [1,2] **(LA)**	-	-	0.632 (0.344–0.795)	0.755 (0.392–0.887)	0.328 (0.042–0.563)

Blue: Excellent (>0.90); Green: Good (0.75–0.90); Yellow: Moderate (0.50–0.75); Red: Poor (<0.50).

**Table 3:** MAPE and MPE between the number of steps recorded by GV4 and manual counting. Variables are reported as mean (CI 95%) for MAPE and mean ± SD for MPE. In HS, data are reported for self-selected speed and slow speed. In PD patients, data are reported for the overall population, in ON, in OFF, and for the most- and least-affected sides. HS-SE: Healthy subjects at self-selected speed; HS-SL: Healthy subjects at slow speed; LA: Least-affected side; MA: Most-affected side; MAPE: Mean absolute percentage error; MPE: Mean percentage error; PD: Parkinson’s disease.

	HS-SE	HS-SL	PD	PD ON	PD OFF	HS-SE vs. PD	ON vs. OFF
**MAPE**	2.38 (1.80–2.95)	8.07 (4.97–11.18)	6.26 (4.48–8.04)	5.53 (3.91–7.16)	7.14 (4.68–9.61))	*p* < 0.001	*p* = 0.047
**MAPE (MA)**	-	-	6.78 (5.06–8.50)	6.04 (4.42–7.66)	7.54 (5.05–10.02)		NS
**MAPE (LA)**	-	-	5.57 (3.54–7.60)	5.03 (3.28–6.78)	6.96 (4.24–9.69)		*p* = 0.018
**MA vs. LA**	-	-	*p* = 0.029	NS	NS		
**MPE**	−0.46 ± 2.98	4.38 ± 11.60	−4.67 ± 7.01	−4.39 ± 6.48	−4.74 ± 9.40		
**MPE (MA)**	-	-	−5.16 ± 7.27	−4.07 ± 7.02	−5.78 ± 9.78		
**MPE (LA)**	-	-	−4.32 ± 7.59	−4.48 ± 6.40	−3.93 ± 10.96		
**MA vs. LA**	-	-	NS	NS	NS		

Blue: Optimal error (<5%); Yellow: Acceptable error (5–10%); Red: Unacceptable error (>10%).

**Table 4:** Spearman rho correlation coefficient between spatiotemporal gait parameters and MAPE in the general population, PD patients, and healthy participants. Significant correlations are reported as follows: * *p* < 0.05; ** *p* < 0.01; *** *p* < 0.001. HS: Healthy subjects; MAPE: Mean absolute percentage error; PD: Parkinson’s disease.

		Distance (m)	Speed (m/s)	Cadence (Steps/min)	Stride Length (m)
**MAPE**	**Overall**	−0.199 *	−0.213 **	−0.048	−0.224 **
	**PD**	0.169	0.106	0.256	−0.018
	**HS**	−0.345 ***	−0.341 ***	−0.367 ***	−0.230 *

## Data Availability

The data presented in this study are available on request from the corresponding author.

## Supplementary Materials

The following supporting information can be downloaded at: https://www.mdpi.com/article/10.3390/s23010214/s1, Table S1: Clinical scores of PD patients; Table S2: Spatiotemporal gait parameters; Table S3: Number of steps measured manually and by GV4.
