# Supplementary material for: Step-Counting Accuracy of a Commercial Smartwatch in Mild-to-Moderate PD Patients and Effect of Spatiotemporal Gait Parameters, Laterality of Symptoms, Pharmacological State, and Clinical Variables
Source: Sensors (Basel). 2022 Dec 25;23(1):214. doi: 10.3390/s23010214 (PMC9823757; doi:10.3390/s23010214)
Supplement: Supplementary file 1 [file sensors-23-00214-s001.zip › sensors-2113600-supplementary.pdf]

Supplementary materials

Supplementary Table S1. Clinical scores of PD patients. Data are reported for the overall PD population and in ON and OFF condition. FIM: Functional Independence Measure; MDS-UPDRS: Movement Disorders Society Unified Parkinson's Disease rating Scale; PD: Parkinson's Disease. PDQ-39: Parkinson's Disease Questionnaire 39; WSCT: Word color Stroop-test;

	**PD**	**PD ON**	**PD OFF**
**MDS-UPDRS-I**	7 (6-11)	-	-
**MDS-UPDRS-II**	7 (4-10)	-	-
**MDS-UPDRS-III**	25 (18 – 33)	21 (16 – 29)	30 (21– 36)
**WCST time (s)**	40.3 ± 16.1	39.5 ± 11.7	40.2 ± 19.8
**WCST errors**	1 (0 – 3)	1 (0 – 3)	1 (0 – 4)
**FIM**	124 (118 – 126)	-	
**PDQ-39**	17 (8 – 28)	-	

Supplementary Table S2. Spatiotemporal gait parameters. Data are reported for healthy participants at both self-selected and slow speed and for the PD patients, in the overall population, in ON and OFF condition. HS-SE: Healthy subjects at self-selected pace; HS-SL: Healthy subjects at slow pace; PD: Parkinson's Disease.

	**HS-SE**	**HS-SL**	**PD**	**PD ON**	**PD OFF**
**Distance (m)**	473 ± 70	287 ± 51	379 ± 73	382 ± 62	382 ± 75
**Speed (m/s)**	1.44 ± 0.22	0.86 ± 0.15	1.15 ± 0.19	1.18 ± 0.20	1.14 ± 0.18
**Cadence (steps/min)**	112 ± 9	83 ± 9	114 ± 10	116 ± 11	113 ± 7
**Stride length (m)**	1.56 ± 0.22	1.24 ± 0.18	1.22 ± 0.20	1.24 ± 0.20	1.23 ± 0.19

Supplementary Table S3. Number of steps measured manually and by GV4. Data are reported for healthy participants at both self-selected and slow speed and for the PD patients, in the overall population, in ON and OFF condition. GV4: Garmin Vivosmart 4; HS-SE: Healthy subjects at self-selected pace; HS-SL: Healthy subjects at slow pace; LA: Least affected side; MA: Most affected side; PD: Parkinson's Disease.

	**HS-SE**	**HS-SL**	**PD**	**PD ON**	**PD OFF**
**Manual**	661 ± 42	493 ± 52	639 ± 56	649 ± 62	637 ± 41
**GV4**	664 ± 46	473 ± 82	667 ± 58	676 ± 62	666 ± 58
**GV4 (MA)**	-	-	670 ± 62	675 ± 62	675 ± 68
**Gv4 (LA)**	-	-	664 ± 58	677 ± 63	659 ± 62
